# Motor Competence Profiles in Greek Primary School Children: A Cross-Sectional Multilevel Analysis of Skill-Specific and Contextual Variability

**DOI:** 10.3390/children13040567

**Published:** 2026-04-19

**Authors:** Andreas Skiadopoulos, Dimitra Dimitropoulou, Theodoros Ellinoudis, Ermioni Katartzi, Christina Evaggelinou

**Affiliations:** Department of Physical Education and Sports Sciences―Serres, Aristotle University of Thessaloniki, 62500 Serres, Greece; ddimit@sch.gr (D.D.); tellinou@phed-sr.auth.gr (T.E.); noni@phed-sr.auth.gr (E.K.); evaggeli@phed-sr.auth.gr (C.E.)

**Keywords:** motor competence, inter-individual variability, contextual environment, multilevel modelling

## Abstract

**Highlights:**

**What are the main findings?**

- Class-level context accounted for a substantial proportion of variance in motor competence, with over half of classes deviating from the population mean.
- Motor competence was domain specific, with balance highest and manual dexterity lowest.

**What are the implications of the main findings?**

- School-based interventions should address between-class heterogeneity in physical education quality.
- Manual dexterity is a priority target for curriculum enrichment in Greek primary physical education.

**Abstract:**

Background/Objectives: Motor competence is a key indicator of children’s developmental readiness and an important component of health and well-being education. It is conceptualized as a latent construct shaped by both individual and contextual factors. The objective of this study was to examine the influence of sex, age and class context on motor competence, with particular emphasis on skill-specific and contextual variability. Methods: Motor competence was assessed in 312 Greek primary school children aged 6–12 years (156 girls) using the Movement Assessment Battery for Children–Second Edition. Standard scores for manual dexterity, aiming–catching, and balance were analyzed using a multilevel modeling approach. Results: Balance showed the highest standard scores, while manual dexterity was the lowest-performing domain. Boys outperformed girls in aiming–catching, with a modest effect. Age effects were domain-specific, with relative age within the classroom negatively associated with manual dexterity but not with other domains. Class-level factors explained substantial variance, indicating heterogeneity across classes. Conclusions: Motor competence in primary school children is strongly domain-specific and meaningfully associated with classroom context. Manual dexterity emerges as a potential priority for curriculum development, and age-related effects appear to operate selectively across domains.

## 1. Introduction

Motor competence, defined as the capacity to combine and scale fundamental movement skills to meet task and environmental demands with adequate proficiency, is a core indicator of developmental health in childhood [1,2]. Early proficiency in fundamental movement skills establishes the foundation for the specialized movement phase of later childhood. Insufficient mastery during the fundamental movement phase constrains this developmental progression and is associated with lower physical activity participation, reduced psychosocial well-being, and poorer health outcomes across the lifespan [3,4,5]. These consequences also affect academic functioning. Motor competence is positively associated with physical activity, which in turn is linked to cognitive and academic performance [6]. These properties have positioned motor competence centrally within global developmental frameworks. Sustainable Development Goal Indicator 4.2.1 identifies motor competence as a primary measure of children’s developmental readiness, while UNESCO and UNICEF recognize it as a foundational component of health and well-being education [7,8,9]. Understanding the sources of variability in motor competence is therefore a public health priority, given its long-term associations with physical fitness, physical activity participation, health-related quality of life, and cognitive and social outcomes across the lifespan [10,11].

Motor competence is a latent construct [1]. It is typically conceptualized as comprising multiple domains, including manual dexterity, aiming–catching, and balance. These domains rely on partially distinct functional and neural systems and may follow different developmental trajectories across childhood [1,11,12,13]. As a result, children often display heterogeneous motor profiles, with strengths in some domains and weaknesses in others. This intra-individual variability can be obscured when motor competence is treated as a single composite score, potentially leading to misleading conclusions regarding developmental status or intervention needs [14,15,16].

In addition to intra-individual variability, inter-individual differences in motor competence have been documented, arising from interactions among biological, environmental, and sociocultural factors [1,11,17]. Sex differences, for example, have been reported in specific domains, with boys often outperforming girls in object control tasks and girls sometimes showing advantages in balance. However, these patterns are inconsistent across studies and may reflect developmental stage or contextual influences rather than stable sex-based differences [12,16,17,18]. This further underscores the need to examine motor competence at the domain level.

Beyond individual-level factors, the educational context may also play an important role. Children in the same classroom share access to similar instructional practices, physical education curricula, and learning environments. These shared experiences may influence motor competence, and the relative development of specific domains. Physical education classes represent, for many children, the primary or sole structured opportunity for motor skill instruction [19], meaning that between-class differences in motor competence outcomes have direct implications for developmental equity. If a substantial proportion of variance in children’s motor competence is attributable to the class they attend rather than to individual biological or demographic factors it implicates modifiable contextual factors such as teacher expertise, class size, curriculum quality, and available resources as intervention targets [11,19]. However, many studies treat observations as independent and do not account for the hierarchical structure of school-based data, potentially underestimating contextual effects and misattributing variance to individual characteristics [18,20].

This limitation is particularly relevant in the Greek context. Previous research suggests that Greek children may exhibit lower motor competence compared to peers in countries with different physical education systems, with variability observed across domains, sex, and age [21,22,23,24,25]. Despite this, no study to date has explicitly modeled class-level contributions to domain-specific motor competence profiles in Greek primary school children.

The present study addresses this gap by assessing motor competence across manual dexterity, aiming–catching, and balance in children aged 6–12 years. A multilevel modeling approach was used to account for the nested structure of the data and to capture both inter-individual differences and contextual variability at the class-level. Three hypotheses were tested: (a) motor competence differs across domains and these differences vary by sex; (b) age is associated with motor competence, but its effect differs across domains; and (c) classroom context contributes meaningfully to both motor competence and domain-specific profiles.

## 2. Materials and Methods

This cross-sectional, comparative study recruited children using a stratified convenience sampling approach by age and gender from 14 elementary schools in western and central Macedonia, Greece. Initially, five regions of Macedonia were randomly selected. Within each region, five elementary schools in urban areas (population > 30 K) and five in semi-urban areas (population < 10 K) were randomly chosen, resulting in a preliminary list of 50 schools. Schools were required to have at least six classes and a minimum of 100 enrolled students to ensure sufficient sample size and classroom diversity. Some schools declined to participate, resulting in a final sample of 14 schools that met all criteria. These schools represented both urban and semi-urban areas, capturing geographical and social diversity.

Within each participating class, four boys and four girls were randomly selected using a computer-generated random number list applied to the class register. Each student was assigned a unique identifier, and random numbers corresponding to eligible students were generated to select participants. Classes unable to provide the full quota of participants were excluded from the sampling frame to preserve the intended stratification. In schools with multiple classes of the same grade and sufficient class size, four boys and four girls were selected from each class. Eligible participants were children aged 6 to 12 years with no diagnosed intellectual, neurological, sensory, or developmental disorders, specific learning difficulties, attention deficit hyperactivity disorder, or chronic illnesses. Children referred for clinical assessment by their schoolteachers were excluded, regardless of formal diagnosis. Written informed consent was obtained from parents or guardians before participation. The final sample included 312 children (156 girls) from 31 classes across 14 schools, with class sizes of either 8 students (*n* = 23 classes) or 16 students (*n* = 8 classes). The target sample size was determined by conventional considerations for multilevel designs, with a minimum of 30 higher-level units (classes) recommended to obtain stable variance component estimates [20]. Post hoc minimum detectable effect sizes at 80% power, estimated via full parametric bootstrap accounting for the two-level random effects structure, confirmed adequate power for the primary outcome (MDES η^2^*p* = 0.058 for the component main effect). The study was approved by the Aristotle University of Thessaloniki Research Ethics Committee and conducted in accordance with the Declaration of Helsinki. Procedures adhered to STROBE guidelines.

Motor competence was assessed by four trained members of the Adapted Physical Education Laboratory using the Greek adaptation of the Movement Assessment Battery for Children–Second Edition (MABC–2) to identify children performing significantly below age–matched peers [26,27]. The MABC–2 is a norm–referenced test for children aged 3–16 years, comprising eight tasks per age band (3–6, 7–10, 11–16 years), organized into three domains: manual dexterity (three items), aiming–catching (two items), and balance (three items assessing static and dynamic balance). Consistent with the study hypotheses, a product-oriented assessment of both fine and gross movement skills was used rather than a process-oriented test to provide descriptive profiles of developmental changes in motor competence among typically developing children [28]. Compared to other product-oriented tests, the MABC–2 offers the advantage of clearly defined age bands, allowing age-appropriate assessment and interpretation of fine and gross motor competence. Raw scores were converted into age–adjusted standard scores, and percentiles were calculated for each domain and for the total score. Pre-established cut-offs were used: standard scores above the 15th percentile were classified as typical performance (green zone); scores between the 6th and 15th percentiles were classified as “at risk” (amber zone); and scores at or below the 5th percentile were classified as significant motor difficulties (red zone). United Kingdom norms of the test as reported in the published manual were used in the present study. Assessments followed the MABC–2 manual and were administered in the school setting. Interrater reliability among the four assessors was monitored using an intraclass correlation coefficient, which for the total score was 0.83 indicating good agreement across raters (ICC; thresholds: <0.50 poor, <0.75 moderate, <0.90 good, >0.90 excellent) [29]. The MABC–2 demonstrates good reliability [26,27,30,31]. Regarding its validity and according to the manual, the MABC–2 covers enough of the validity conditions. Moreover, the manual supports the view that the motor test is culture-neutral [27]. Its cross-cultural validity has also been supported by many studies, showing that the MABC–2 is considered a reliable and useful tool in identifying children with motor deficiencies [32].

Standard scores from the MABC–2 were analyzed using a linear mixed-effects model fitted by Restricted Maximum Likelihood (REML), implemented in the nlme package [33]. Initial fixed effects included component (manual dexterity, aiming–catching, balance), grand mean-centering age, sex, and their interactions. Categorical predictors were sum-coded to support Type III inference. Model selection proceeded sequentially via likelihood ratio testing using maximum likelihood (ML) refits: a model with class-level domain-specific random slopes fitted significantly better than one with class-level random intercepts only (LRT χ^2^(5) = 112.04, *p* < 0.001); a school-level random intercept did not improve fit (LRT χ^2^(1) = 0.03, *p* = 0.859) and was excluded; the three-way component × age × sex interaction and non-significant two-way terms were dropped without significant loss of fit (LRT χ^2^(3) = 1.57, *p* = 0.667); and the component × age interaction was subsequently excluded (LRT χ^2^(2) = 4.26, *p* = 0.119). To determine whether the age effect reflected a suppression artefact arising from the substantial between-class variation in mean age, age was decomposed into a within-class component (each student’s deviation from their class mean age) and a between-class component (class mean age), following the contextual effects modelling approach [34]. This decomposition allows the within-class and between-class effects of age to be estimated independently. The decomposed model fitted significantly better than combined age (grand mean-centering age) (LRT χ^2^(1) = 6.34, *p* = 0.012, ΔAIC = −4.3) and was adopted as the final model. The final parsimonious model retained component, within-class age, between-class age, sex, and component × sex as fixed effects.

To capture intra-individual variability in domain-specific motor profiles, correlated domain-specific random slopes were modelled at the student level under a general positive-definite covariance structure (Log-Cholesky parametrization), allowing students to differ both in overall motor competence (random intercept) and in the shape of their domain profiles across aiming–catching and balance (random slopes). To capture contextual variability at the class level, correlated domain-specific random slopes were similarly modelled at the class level, allowing classes to differ both in overall motor level and in their domain-specific profiles, reflecting heterogeneity in the instructional emphasis of individual classrooms. This two-level random slope structure, at both the student and class levels, was supported by likelihood ratio testing and by the theoretical expectation that contextual class-level effects operate differentially across motor domains.

Residual homoscedasticity was confirmed using the Breusch–Pagan test (*p* = 0.161; performance package), and multicollinearity was assessed via generalized Variance Inflation Factors, with all values ≤1.03, indicating negligible collinearity among predictors. Fixed effects were tested using Type III Wald F-tests (α = 0.05). Effect sizes were quantified as partial η^2^ (η^2^p), computed via the effectsize package and interpreted per Cohen’s benchmarks [35]. Post hoc pairwise contrasts were conducted as general linear hypotheses via the multcomp package, with familywise error rate controlled using the Westfall correction [36]. Effect sizes for pairwise contrasts were expressed as Cohen’s d, computed from the model t-statistics and degrees of freedom as implemented in the lme.dscore function from the EMAtools package, which standardizes effects against the total multilevel variance pooled across class-level, individual-level, and residual components [37]. Post hoc power analysis was conducted via full parametric bootstrap (200 iterations per effect size) using custom simulation code. At each iteration, new response data were generated from the fitted model by sampling class-level and student-level random effects from their estimated multivariate normal distributions (3 × 3 covariance matrices at each level), computing the fixed-effect prediction under a specified effect size, and adding residual noise. The model was refit and the *p*-value and η^2^p extracted at each iteration. This procedure was repeated across a range of fixed-effect coefficient values to generate power curves for each fixed effect, from which the minimum detectable effect size (MDES) at 80% power was interpolated as the median η^2^p across significant iterations at the 80% power threshold. This procedure additionally served as a sensitivity analysis for statistical power: by comparing observed effect sizes against their MDES, it was possible to determine whether non-significant findings reflected negligible effects or insufficient power to detect effects of meaningful magnitude, and whether significant findings exceeded or fell below the detectable threshold, indicating possible upward bias (Type M error). In such cases, the observed effect size is reported alongside the MDES to contextualize its likely precision, and the finding is flagged as requiring replication in larger samples. No missing data were present in the dataset analyzed. All 312 enrolled participants contributed complete data across all three MABC-2 component scores, age, and sex, yielding 936 observations for analysis. All analyses were performed in R version 4.5.2 [38]. Anthropic’s Claude AI Sonnet 4.6 has been used in this paper to assist in code generation and result interpretation.

## 3. Results

**Figure 1:**
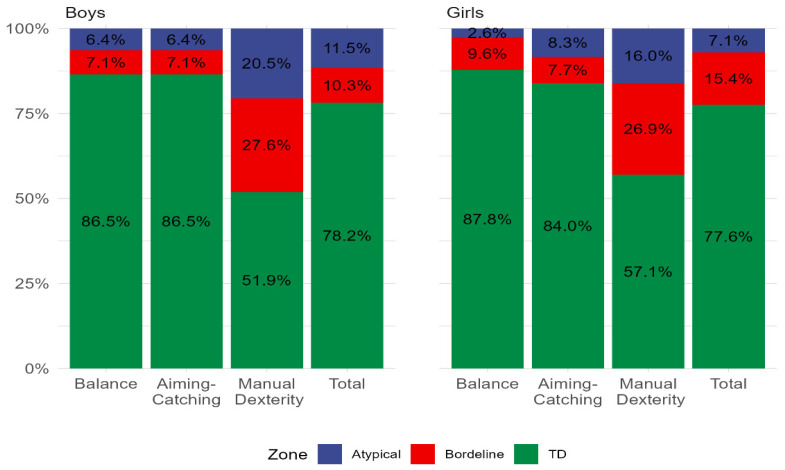
Percentage of boys and girls classified by MABC-2 percentile ranges across domains (manual dexterity, aiming–catching, balance) and total score. Percentile ranges shown correspond to typical performance (>15th percentile; TD), at risk (6th–15th percentile; Borderline), and severe motor coordination difficulties (≤5th percentile; Atypical).

**Figure 2:**
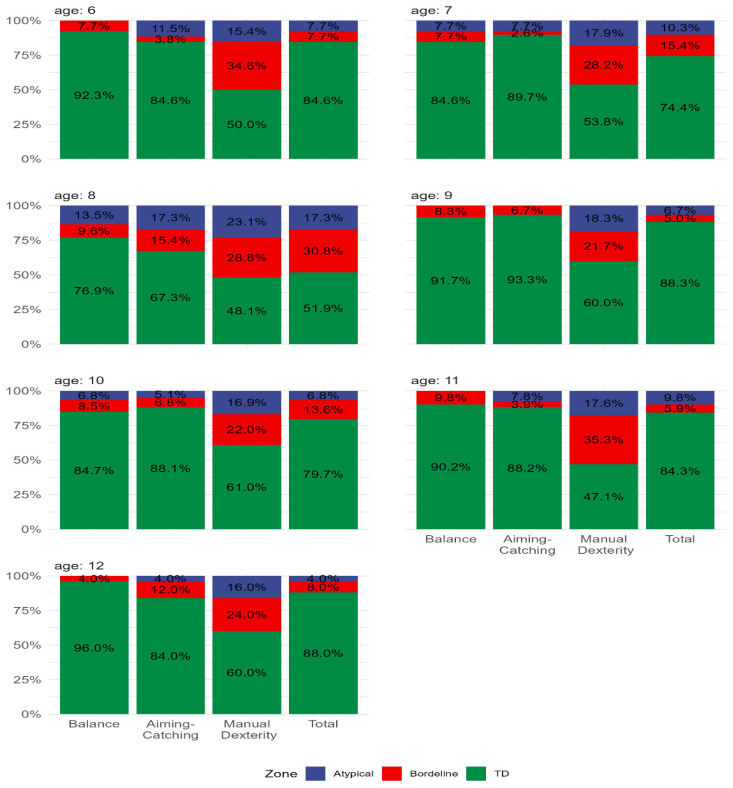
Percentage distribution of children aged 6–12 years classified as typical performance (>15th percentile; TD), at risk (6th–15th percentile; Borderline), and severe motor coordination difficulties (≤5th percentile; Atypical) across motor domains (manual dexterity, aiming–catching, balance) and the total score.

**Figure 3:**
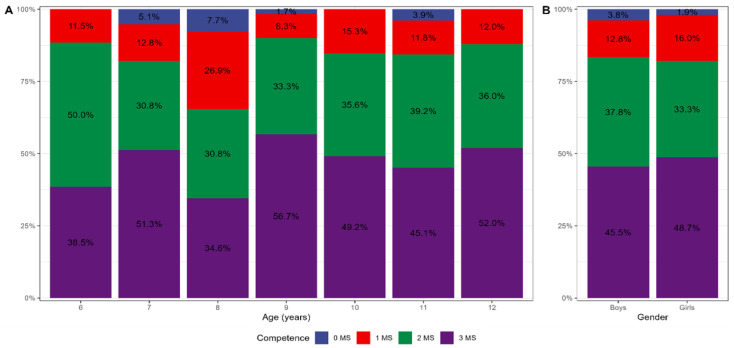
(**A**) Proportion of children achieving competence in none (0 MS), one (1 MS), two (2 MS), or all three (3 MS) MABC–2 skills components (manual dexterity, aiming–catching, balance) by age. (**B**) Distribution of boys and girls achieving competence in none (0 MS), one (1 MS), two (2 MS), or all three (3 MS) MABC-2 skills components (manual dexterity, aiming–catching, balance).

All 312 enrolled participants provided complete data. For the whole sample, approximately 13% were classified as “at risk” for movement difficulties (≤16th percentile), with 9% at or below the 5th percentile indicating severe motor coordination difficulties. The percentages of boys and girls across MABC-2 percentile ranges for manual dexterity, aiming–catching, balance, and total scores are presented in Figure 1. Overall, 10.3% of boys and 15.4% of girls were classified as “at risk” (amber zone), while 11.5% of boys and 7.1% of girls were classified in the red zone (at or below the 5th percentile), reflecting marked inter-individual variability in motor competence risk profiles across sex. Figure 2 shows percentile distributions for manual dexterity, aiming–catching, and balance across ages six to 12 years. Descriptively, manual dexterity showed the lowest percentile scores across all age groups. Figure 3A shows age-specific mastery in none, one, two, or all three MABC-2 components. The proportion of children achieving competence in all three components was 38.5% at age six and 34.6% at age eight. Conversely, the proportion failing to achieve competence in any component was 5.1% at age seven, 7.7% at age eight, 1.7% at age nine, and 3.9% at age eleven, reflecting substantial inter-individual variability in overall motor competence across the age range. Figure 3B shows the distribution of boys and girls achieving competence in none, one, two, or all three MABC-2 components. Table 1 shows mean MABC-2 scores and component scores across sex and age.

Component scores differed substantially across manual dexterity, aiming–catching, and balance (F(2, 620) = 48.77, *p* < 0.001, η^2^p = 0.14 [0.09, 0.19], MDES = 0.058), confirming adequate power for the primary outcome. The grand mean-centering age fixed effect in the original model specification was non-significant (F(1, 279) = 0.015, *p* = 0.901, η^2^p < 0.001). However, this null finding reflected a suppression effect probably arising from the substantial between-class variation in mean age (SD = 1.71 years). Following decomposition into within-class and between-class components, within-class age was significantly associated with motor competence standard scores (F(1, 279) = 4.96, *p* = 0.027, η^2^p = 0.02 [0.00, 0.06], β = −0.495, SE = 0.223, MDES = 0.028), while between-class age was not (*F*(1, 29) = 1.67, *p* = 0.207, η^2^p = 0.05 [0.00, 0.27], β = 0.132, SE = 0.102, MDES = 0.284). The negative within-class gradient indicates that children who are older relative to their classmates score lower than their younger peers. Supplementary analyses confirmed that this effect was not attributable to artefacts from mixing MABC–2 age-bands within classrooms: it did not differ between single-band and multi-band classes (F(1, 278) = 0.09, *p* = 0.765), was not moderated by age-band membership (F(2, 275) = 1.73, *p* = 0.179), and remained significant when band-boundary classes were explicitly modelled (F(1, 278) = 4.65, *p* = 0.032). Consequently, the interaction component × within-class age was tested in the model, and it was significant (F(2, 618) = 3.13, *p* = 0.044), indicating that age effects differed across motor domains. Examination of domain-specific slopes revealed that the negative association between age and motor competence was primarily observed in manual dexterity (β = −0.90, 95% CI [−1.45, −0.35]), whereas no significant age effects were found for aiming–catching or balance. However, comparisons of slopes did not reach statistical significance after adjustment (*t*(618) = 2.33, *p*adj = 0.052), suggesting that differences between domains should be interpreted cautiously.

Sex (F(1, 279) = 0.184, *p* = 0.668, η^2^p < 0.001 [0.00, 0.00]) did not produce a significant main effect, with observed effect far below its MDES threshold (observed η^2^p < 0.001 vs. MDES η^2^p = 0.026), indicating that a global sex effect on motor competence, if present, is negligibly small in this population rather than undetected due to insufficient power. A significant component × sex interaction was found (F(2, 620) = 9.47, *p* < 0.001, η^2^p = 0.03 [0.01, 0.06], MDES = 0.094), indicating that sex differences in motor competence were domain-specific. However, the observed effect fell 3.2 times below its MDES threshold, indicating potential upward bias. When significant effects fall below the MDES threshold, their direction may be considered reliable, but their magnitude should be interpreted cautiously, as it is likely an overestimate of the true population effect (Type M error [39]). Accordingly, this finding warrants cautious interpretation pending replication in larger samples.

Pairwise comparisons of component estimated marginal means, collapsed across sex, confirmed that all three components differed significantly (all *p*adj ≤ 0.043), with balance yielding the highest scores, followed by aiming–catching and manual dexterity. The contrasts involving manual dexterity were medium–large in magnitude (balance vs. manual dexterity: d = 0.759; aiming–catching vs. manual dexterity: d = 0.985), while balance exceeded aiming–catching by a smaller but significant margin (d = 0.242, *p*adj = 0.043). Stratified by sex at mean age, the balance superiority over manual dexterity was preserved for both boys (d = 1.37) and girls (d = 1.66), as was the aiming–catching superiority over manual dexterity (boys: d = 1.16; girls: d = 0.56), all large-to-medium effects consistent with the overall inter-individual ordering of domains. One critical sex-specific difference emerged in the inter-individual profile: balance and aiming–catching did not differ among boys (*t*(620) = 0.18, *p*adj = 0.855, d = 0.03) but differed substantially among girls (*t*(620) = 3.36, *p*adj = 0.001, d = 0.37), with girls showing balance superiority over aiming–catching that was absent in boys. Examining sex differences within each component at mean age revealed that inter-individual sex differences were domain-specific: boys scored significantly higher than girls only on aiming–catching (*t*(279) = 3.15, *p*adj = 0.002, d = 0.38), a small-to-medium effect. No significant sex differences emerged for balance (*t*(279) = −0.72, *p*adj = 0.475, d = −0.09) or manual dexterity (*t*(279) = −1.86, *p*adj = 0.064, d = −0.22). Given that the omnibus component × sex interaction effect size fell below its MDES threshold (η^2^p = 0.03, MDES = 0.094), all component × sex contrasts should be interpreted cautiously as potentially subject to upward bias (Type M error [39]).

The progressive model comparison confirmed that both the class-level random intercept and class-level domain-specific random slopes were statistically justified (Table 2). The final model fitted significantly better than a null model with the same random effects structure (LRT χ^2^(6) = 63.37, *p* < 0.001, Nagelkerke pseudo-R^2^ = 0.07), where the modest Nagelkerke value reflects the incremental contribution of fixed effects over and above the domain-specific profiles already captured by the random effects. Fixed effects alone explained 24% of total variance (R^2^marginal = 0.24), while the full model accounted for 92% (R^2^conditional = 0.92) [40], with the difference (~69%) attributable to systematic inter-individual and contextual between-class variability in domain-specific motor profiles. The variance partition coefficient at the grand mean was 0.22, indicating that 22% of the variability in motor competence scores was attributable to contextual class membership net of fixed effects. Across all variance components, contextual class-level sources collectively accounted for 23.1% of total variance, comprising between-class differences in overall motor level (8.9%), aiming–catching profiles (7.9%), and balance profiles (6.3%). The dominant source of variance was inter-individual variability at the student level (69.1%), reflecting large differences in overall motor performance (25.4%), aiming–catching profiles (21.2%), and balance profiles (22.4%), with residual within-student variance contributing 7.8% (σ = 0.95). The substantial intra-individual variability in domain-specific profiles―reflected in the large student-level slope variances―confirms that individual children’s relative strengths and weaknesses across motor domains vary considerably around the population average. Empirical Bayes class-level estimates revealed that 17 of 31 classes (52%) deviated significantly from the grand mean on the overall motor intercept (|intercept| > 1.96 × posterior SE), underscoring the extent of contextual between-class heterogeneity.

**Figure 4:**
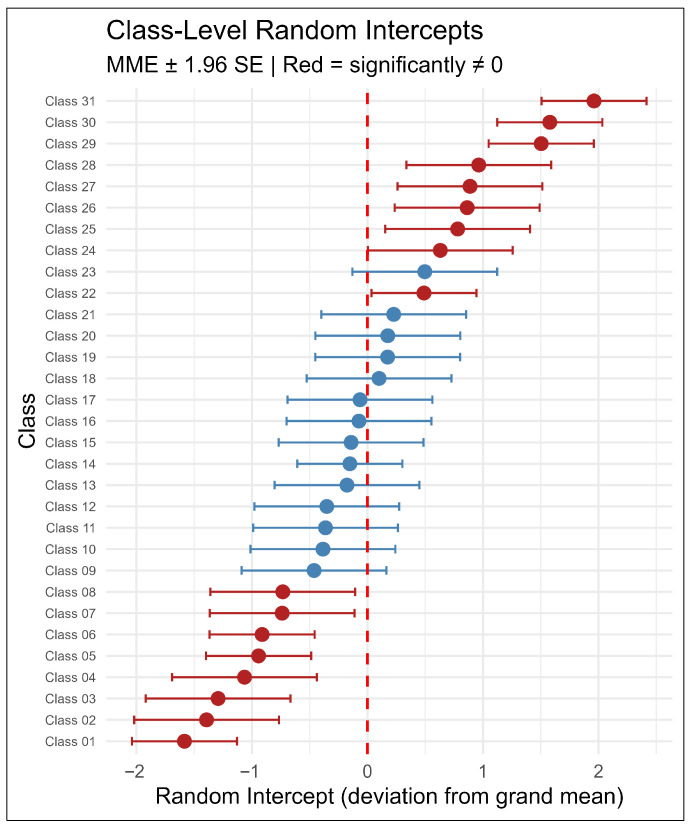
Caterpillar plot of the random effects. Horizontal lines indicate 95% prediction intervals for classes.

Class intercept deviations ranged from −1.58 to 1.96 standard score points (Figure 4). Contextual domain-specific slope deviations at the class level ranged from −1.63 to 1.82 for aiming–catching and from −1.53 to 2.30 for balance, indicating substantial heterogeneity in domain profiles across classrooms beyond what was explained by fixed effects. Class-level intercepts were positively correlated with both domain slopes (aiming–catching: r = 0.49; balance: r = 0.35), indicating that contextually higher-performing classes tended to show uniformly elevated profiles across all domains. A mild negative correlation between domain slopes (r = −0.26) suggested a weak contextual trade-off between aiming–catching and balance emphasis at the class level. At the student level, a strong negative correlation between domain slopes (r = −0.58) reflected pronounced intra-individual specialization: students with stronger aiming–catching profiles consistently showed comparatively flatter balance profiles, a pattern substantially more pronounced than the corresponding contextual class-level trade-off, suggesting that intra-individual domain specialization operates primarily within rather than across classrooms.

## 4. Discussion

The present cross-sectional study examined motor competence in Greek primary school children across three functionally distinct domains: manual dexterity, aiming–catching, and balance. A multilevel modeling framework was used to explicitly partition inter-individual and contextual sources of variability. Three main findings emerged. First, motor competence was domain-specific, with balance showing the highest scores and manual dexterity the lowest. Second, sex differences were also domain-specific rather than global, with boys outperforming girls in aiming–catching, although this effect should be interpreted cautiously. Third, classroom context accounted for a substantial proportion of variance in motor competence, with marked heterogeneity across classes in both overall performance and domain-specific profiles.

The finding that balance yielded the highest standard scores and manual dexterity the lowest, with all three domains differing significantly, aligns with the multidimensional structure of motor competence as assessed by the MABC-2 [27] and with evidence that the three domains recruit partially dissociable neural substrates and show non-parallel developmental trajectories. Approximately 13% of children were classified as “at risk” (≤16th percentile) and 9% showed scores consistent with severe coordination difficulties (≤5th percentile), proportions broadly comparable to international prevalence estimates for developmental coordination disorder (DCD) of 5–8% [41]. However, while low motor competence is one diagnostic criterion for DCD, it should not be conflated with DCD. Some children with limited exposure to skill learning may temporarily present with low motor competence but demonstrate rapid improvement when given targeted opportunities. Greek children’s performance on combined fine and gross motor assessments has previously been found to be lower than that of Norwegian peers, with reduced physical education exposure proposed as a contributing contextual factor [22]. The present findings extend this literature by demonstrating that within-sample domain differences are large and that manual dexterity specifically emerges as the most consistently low-performing domain across the sample. From a public health perspective, this pattern is directly relevant to SDG Indicator 4.2.1: children performing below age expectations on fine motor tasks may face constraints not only in sport and physical activity but in the acquisition of academic skills dependent on fine motor precision, including writing and tool use [42].

It should be noted, however, that these classification rates are based on UK normative data. Although this approach is standard in the absence of locally validated norms, normative samples are inherently population-specific and may not fully capture the distribution of motor performance in different cultural contexts. Motor development is increasingly conceptualized as an enculturated process shaped by culturally specific activity patterns and environmental affordances [43,44,45], and empirical evidence demonstrates systematic cross-cultural differences in MABC-2 performance [46,47]. Within this framework, applying UK-derived cut-off scores to a Greek sample may lead to misestimation of motor difficulty prevalence, with potential over- or under-identification depending on domain-specific deviations from the reference distribution. This limitation primarily affects absolute classification rates and cross-cultural comparisons and should therefore be considered when interpreting prevalence estimates. In contrast, the multilevel analyses reported here are based on within-sample variability and relative differences across children and classes; thus, the observed associations between predictors and motor performance are unlikely to be materially influenced by the choice of normative framework.

Hypothesis (a) was partially supported. Boys showed a significantly higher aiming–catching performance than girls, a small-to-medium effect consistent with the sex-by-domain interaction in the motor competence literature, where boys outperform girls on object control tasks [11,48]. This sex difference in aiming–catching is typically attributed to differential exposure to ball-sport activities during leisure time and physical education, as well as to sociocultural expectations that channel boys and girls differently toward object control versus rhythmic or balance-focused activities [49,50]. The predicted female advantage in manual dexterity did not reach statistical significance, consistent with the non-significant main effect of sex, suggesting that any female advantage in fine motor performance in this population, if present, was too small to detect with the current sample size or was obscured by the large inter-individual variability in manual dexterity scores within both sexes.

A particularly informative finding was the sex-specific pattern within the balance vs. aiming–catching contrast. Balance substantially exceeded aiming–catching among girls but not among boys, indicating that the domain ordering of scores differed qualitatively by sex. Girls showed the theoretically expected balance superiority over aiming–catching that reflects girls’ relative advantage in dynamic balance tasks and relative disadvantage in object control [11], whereas boys’ balance and aiming–catching performance were statistically indistinguishable. This finding suggests that the intra-individual motor profile is sex-differentiated in ways that aggregate comparisons of overall motor competence would conceal. These results reinforce the methodological importance of analyzing motor competence at the domain level rather than as a single composite. However, because the component × sex interaction fell below its MDES threshold it should be interpreted as preliminary patterns requiring replication in larger samples before informing targeted sex-specific interventions. The absence of a significant sex main effect indicates that global sex differences in overall motor competence were negligibly small in this population. This finding is consistent with studies emphasizing that sex differences in motor competence are domain-specific rather than reflecting a generalized advantage for either sex [11,48] and argues against educational practices that treat boys and girls as categorically different in overall motor ability.

Hypothesis (b) was supported. A significant interaction between component and within-class age indicated that age effects varied across motor domains. Specifically, older children relative to their classmates showed lower performances in manual dexterity, whereas no meaningful age-related differences were observed in aiming–catching or balance. This pattern suggests that relative age effects operate selectively, influencing fine movement skills more strongly than other motor domains. One possible explanation is that manual dexterity tasks are more sensitive to relative performance expectations within the classroom context, although this interpretation remains speculative and requires further investigation.

Hypothesis (c) was fully supported. The class context accounted for 23.1% of total variance in motor competence, with the class-level random intercept and domain-specific random slopes both statistically justified by likelihood ratio testing. This finding is consistent with the bioecological model of human development [51] and with multilevel evidence from educational research indicating that class-level factors are associated with developmental outcomes beyond individual-level characteristics [18,52]. However, the mechanisms underlying this association in the present study cannot be determined from the available data, as no class-level covariates were measured. Theoretically relevant class-level variables include physical education curriculum characteristics (e.g., lesson frequency, activity type, and degree of instructional structure), teacher-related factors (e.g., specialist versus generalist status, teaching experience, and motivational orientation), class size, and the availability and variability of physical resources and play equipment [19]. Critically, classes differed not only in overall motor level (class intercept variance = 9.3% of total) but in their domain-specific profiles, with the relative variation across classes in aiming–catching and balance profiles accounting for a further 7.8% and 6.3% of total variance respectively. The positive correlation between class-level intercepts and domain slopes indicates that classes with higher overall motor competence tended to show uniformly elevated profiles across all domains. A negative correlation between domain slopes at the class level suggests that classes with relatively stronger aiming–catching profiles tended toward comparatively flatter balance profiles. Class-level data are needed to be collected to test this directly. It is important to emphasize that the random effect for class documents that classroom membership is systematically associated with motor competence outcomes but cannot identify the mechanisms that produce this clustering. These remain a primary target for future research.

The finding that more than the half classes deviated significantly from the grand mean on the overall motor intercept confirms that a child’s motor competence is meaningfully associated with which class they attend, above and beyond their individual characteristics. Classes deviating substantially below the grand mean may represent priority targets for further investigation. However, identifying the specific contextual factors associated with low performance in these classes would require prospective data collection including class-level covariates (e.g., physical education quality, teacher qualifications, facility resources, instructional time) before intervention recommendations can be made [8,9].

The dominant source of variance in motor competence was inter-individual variability at the student level, comprising large individual differences in overall motor level, aiming–catching profiles, and balance profiles. These magnitudes are consistent with dynamical systems theories of motor development, which emphasize that motor competence emerges and stabilizes through exploration, practice, and the continuous interaction of individual, environmental, and task constraints rather than as a hardwired neuromaturational outcome [53,54]. The large inter-individual variability in overall motor competence level is consistent with dynamical systems theories attributing individual differences in motor competence to different developmental timescales [14,15]. The large intra-individual variability in domain-specific profiles (reflected in the student-level slope variances for aiming–catching and balance and their strong negative correlation) further indicates that individual children’s relative strengths and weaknesses across motor domains vary considerably around the population average. This strong negative intra-individual correlation between aiming–catching and balance slopes suggests a domain specialization pattern: children who perform relatively well on aiming–catching relative to their own overall motor level tend to show comparatively flatter balance profiles, and vice versa. This specialization was substantially more pronounced at the individual level than at the class level, suggesting it reflects individual-level developmental history and practice exposure rather than contextual instructional emphasis. A dynamical systems interpretation is that children’s motor competence profiles represent attractor states that emerge from the specific constellation of affordances, practice opportunities, and task demands they have encountered [53,55]. Children with greater exposure to object control activities may develop more stable aiming–catching coordination patterns, whereas children with greater exposure to locomotor tasks may show stronger balance profiles, though prospective designs measuring individual activity exposure are required to test this hypothesis directly.

The proportion of children failing to achieve competence in any of the three MABC-2 components ranged from 1.7% to 7.7% across age groups, while between 34.6% and 38.5% achieved competence in all three at ages six and eight, respectively, reflecting the substantial inter-individual variability in overall developmental readiness within each age cohort. These figures are relevant to SDG Indicator 4.2.1 monitoring, as they indicate that a meaningful minority of children in Greek primary schools are not developmentally on track in the health domain of motor competence, with potential downstream implications for physical activity participation and health-related outcomes across the lifespan [5].

Several limitations constrain the interpretation of these findings. Cross-sectional design prevents causal inference and precludes tracking of intra-individual developmental trajectories across the elementary school years. The observed domain profiles and between-class differences reflect a single time point and may not generalize to other cohorts or contexts. The class-level variance components reported in the present study may partly reflect shared school-level characteristics that could not be disentangled from class-specific effects given the available sample of schools. Future studies should include a minimum of 30 schools to enable reliable school-level inference and to distinguish between school-level and class-level contributions to motor competence profiles. The cluster size of eight students per class, while sufficient for unbiased fixed-effect estimation may limits the precision of class-level variance component estimates. These should therefore be interpreted as approximate rather than precise population estimates. To the extent that selection bias was introduced by excluding classes unable to provide the full quota of eight children, this may have affected the class-level variance estimates: if excluded classes were systematically lower-performing or more homogeneous, for example because they had fewer enrolled students, the observed between-class heterogeneity (0.22) may be a conservative underestimate of true contextual variability in the population. Conversely, if excluded classes were atypically high-performing, the prevalence of at-risk classification (13%) could be overestimated. The direction of this bias cannot be determined without data from excluded classes and should be acknowledged when interpreting the class-level findings. The absence of contextual covariates, including socioeconomic status, school resources, physical education curriculum content, teacher qualifications, and detailed urban–rural classification, means that the mechanisms underlying the observed class-level heterogeneity cannot be identified. The variance attributed to the class level represents the net effect of all unmeasured contextual factors that classes share, and residual confounding by these factors cannot be excluded. Future studies should therefore incorporate measured class-level covariates, such as teacher characteristics, physical education curriculum features, class size, and facility quality [19] to move beyond documenting contextual variance toward explaining its sources. To the extent that unmeasured socioeconomic or resource variables are associated with both class assignment and motor competence, the fixed effects for age and sex may be subject to omitted variable bias. However, given that both effects were negligibly small (η^2^p < 0.001) and far below their MDES thresholds, this is unlikely to alter the substantive conclusion that global age and sex effects are negligible in this population. Interrater reliability was reported as an overall agreement metric (ICC = 0.83). Future work should report domain-specific reliability using weighted Kappa or absolute ICC with confidence intervals to determine whether reliability varies across motor domains. Finally, MABC-2 standard scores are referenced against United Kingdom norms, which may not fully reflect the developmental expectations of the Greek educational context. Any systematic cultural difference in normative performance would shift prevalence estimates uniformly across the sample without affecting the relative domain ordering or variance partition results.

## 5. Conclusions

In a cross-sectional sample of Greek primary school children, motor competence profiles were domain-specific, with balance exceeding aiming–catching and manual dexterity emerging as the weakest domain. Sex differences were confined to aiming–catching, with boys outperforming girls, while no significant overall sex or age effects were detected. Age effects were domain-specific, with relative age within the classroom negatively associated with manual dexterity but not with other motor domains. The contextual class environment accounted for a substantial proportion of variance and shaped not only the overall level but the domain-specific structure of children’s motor competence profiles, with a large proportion of classes deviating from the population average.

These findings highlight manual dexterity as a potential curricular target in Greek primary school physical education, underscore the importance of modelling motor competence at the domain level rather than as a single composite, and emphasize the need for contextually sensitive school-based interventions that address between-class heterogeneity in motor competence outcomes, the sources of which remain to be identified through future research incorporating class-level covariates. Longitudinal designs incorporating contextual covariates are required to distinguish transient low performance from persistent developmental motor difficulties and to clarify the mechanisms underlying the observed class-level specialization in domain profiles.

## Figures and Tables

**Table 1:** Mean (standard deviation, SD) MABC–2 total and component standardized scores by age and gender.

		6 Years			7 Years			8 Years			9 Years			10 Years			11 Years			12 Years		
		*n* = 26			*n* = 39			*n* = 52			*n* = 60			*n* = 59			*n* = 51			*n* = 25		
		M ± SD	M_d_	CI	M ± SD	M_d_	CI	M ± SD	M_d_	CI	M ± SD	M_d_	CI	M ± SD	M_d_	CI	M ± SD	M_d_	CI	M ± SD	M_d_	CI
**C1**	B	11.7 ± 1.9	11	10.5–12.5	12.6 ± 4.7	16	10.5–16	9.4 ± 4.1	9	8–11	10.8 ± 2.7	11	10–11.5	11.0 ± 2.7	12	11–12.5	12.2 ± 2.7	14	11–14	10.6 ± 2.9	10	8.5–12.5
	G	11.9 ± 2.9	14	10–14	11.9 ± 4.1	12	9.5–14	10.0 ± 2.7	10	8.5–11	12.5 ± 2.0	14	11.5–14	10.8 ± 3.4	12	9.5–12.5	11.6 ± 2.8	12	10.5–12.5	11.3 ± 2.2	10	10–12
**C2**	B	10.3 ± 3.5	11	8–12.5	11.1 ± 3.5	12	9.5–13	9.7 ± 3.5	10	8.5–11	11.3 ± 2.2	11	10.5–12	11.4 ± 3.2	12	10–13	12.5 ± 2.9	13	11.5–13.5	11.8 ± 2.4	12	10–13.5
	G	9.7 ± 3.1	10	8–11.5	10.5 ± 2.0	11	9–12	8.2 ± 3.2	9	7–10	10.9 ± 2.3	11	10–12	10.0 ± 2.1	10	9–10.5	11.0 ± 4.0	10	9–13	11.3 ± 3.9	11	9–13.5
**C3**	B	8.0 ± 2.5	7	6.5–10	7.1 ± 2.4	8	6–8	6.9 ± 2.2	7	6–8	7.4 ± 2.7	8	6.5–8.5	7.7 ± 2.6	8	6.5–8.5	7.3 ± 2.5	7	6.5–8.5	7.1 ± 2.9	8	5–9
	G	7.8 ± 2.5	8	6–9	7.8 ± 2.7	8	6.5–9	6.9 ± 2.2	7	6–8	8.4 ± 1.7	9	7.5–9	8.0 ± 2.3	8	7–9	7.5 ± 2.6	7	6.5–8.5	7.9 ± 2.1	8	6.5–9

Note. Effect sizes (η^2^p, Cohen’s d) and degrees of freedom are reported in–text. C1: Balance; C2: Aiming–catching; C3: Manual dexterity.

**Table 2:** Random effects models comparison.

	df	AIC	BIC	logLik	Test	L. Ratio	*p*-Value
Model 1	14	4574.8	4642.6	−2273.4			
Model 2	15	4548.8	4621.4	−2259.4	1 vs. 2	28.02	<0.0001
Model 3	20	4446.8	4543.6	−2203.4	2 vs. 3	112.04	<0.0001
Final model	21	4442.4	4544.1	−2200.2	3 vs. 4	6.34	=0.0118

Nlme structure: Model 1: random = list (students = ~components) Model 2: random = list (class = ~1, students = ~components) Model 3: random = list (class = ~components, students = ~components) Final model: random = list (class = ~components, students = ~components); df = degrees of freedom; AIC = Akaike Information Criterion; BIC = Bayesian Information Criterion; logLik = Log-likelihood; L. Ratio = Log-Likelihood Ratio.

## Data Availability

The original data presented in the study are openly available in https://figshare.com/s/2e3a808ebad56446a963 (accessed on 14 April 2026).

## Abbreviations

The following abbreviations are used in this manuscript:

MABC-2	Movement Assessment Battery for Children—Second Edition
MDES	Minimum detectable effect size

## References

[1] Martins C., Valentini N.C., Sääkslahti A., Africa E.K., Webster E.K., Nobre G., Robinson L.E., Duncan M., Tortella P., Bandeira P.F. (2024). Motor Competence as Key to Support Healthy Development of 3- to 5-Year-Old Children: An Expert Statement on Behalf of the International Motor Development Research Consortium. J. Mot. Learn. Dev..

[2] Utesch T., Bardid F., Hackfort D., Schinke R., Strauss B. (2019). Motor Competence. Dictionary of Sport Psychology: Sport, Exercise, and Performing Arts.

[3] Brian A., Getchell N., True L., De Meesdter A., Stodden D.F. (2020). Reconceptualizing and Operationalizing Seefeldt’s Proficiency Barrier: Applications and Future Directions. Sports Med..

[4] Clark J.E., Metcalfe J.S. (2002). The Mountain of Motor Development: A Metaphor.

[5] Stodden D.F., Pesce C., Zarrett N., Tomporowski P., Ben-Soussan T.D., Brian A., Abrams T.C., Weist M.D. (2023). Holistic Functioning from a Developmental Perspective: A New Synthesis with a Focus on a Multi-Tiered System Support Structure. Clin. Child Fam. Psychol. Rev..

[6] Haapala E.A., Väistö J., Lintu N., Westgate K., Ekelund U., Poikkeus A.-M., Brage S., Lakka T.A. (2017). Physical Activity and Sedentary Time in Relation to Academic Achievement in Children. J. Sci. Med. Sport.

[7] UNICEF (2023). The Early Childhood Development Index 2030: A New Measure of Early Childhood Development.

[8] UNESCO, UNICEF (2024). Building Strong Foundations: What to Teach for Foundational Education for Health and Well-Being.

[9] UNESCO, UNICEF (2024). Building Strong Foundations: What Is Foundational Education for Health and Well-Being?.

[10] Lubans D.R., Morgan P.J., Cliff D.P., Barnett L.M., Okely A.D. (2010). Fundamental Movement Skills in Children and Adolescents. Sports Med..

[11] Barnett L.M., Lai S.K., Veldman S.L.C., Hardy L.L., Cliff D.P., Morgan P.J., Zask A., Lubans D.R., Shultz S.P., Ridgers N.D. (2016). Correlates of Gross Motor Competence in Children and Adolescents: A Systematic Review and Meta-Analysis. Sports Med..

[12] Assaiante C. (1998). Development of Locomotor Balance Control in Healthy Children. Neurosci. Biobehav. Rev..

[13] Sobinov A.R., Bensmaia S.J. (2021). The Neural Mechanisms of Manual Dexterity. Nat. Rev. Neurosci..

[14] Draper C.E., Yousafzai A.K., McCoy D.C., Cuartas J., Obradović J., Bhopal S., Fisher J., Jeong J., Klingberg S., Milner K. (2024). The next 1000 Days: Building on Early Investments for the Health and Development of Young Children. Lancet.

[15] Lake A., Chan M. (2015). Putting Science into Practice for Early Child Development. Lancet.

[16] Utesch T., Bardid F., Büsch D., Strauss B. (2019). The Relationship Between Motor Competence and Physical Fitness from Early Childhood to Early Adulthood: A Meta-Analysis. Sports Med..

[17] Palmer K.K., Stodden D.F., Terlizzi B.M., Pennell A., Nunu M.A., Robinson L.E. (2025). Motor Development in Early Childhood (3–5 Year Olds): Investigating Longitudinal Changes in Children’s Movement Patterns and Outcome Performance. J. Mot. Learn. Dev..

[18] Goldstein H., Burgess S., McConnell B. (2007). Modelling the Effect of Pupil Mobility on School Differences in Educational Achievement. J. R. Stat. Soc. Ser. A Stat. Soc..

[19] Flôres F.S., Rodrigues L.P., Copetti F., Lopes F., Cordovil R. (2019). Affordances for Motor Skill Development in Home, School, and Sport Environments: A Narrative Review. Percept. Mot. Skills.

[20] Maas C.J.M., Hox J.J. (2005). Sufficient Sample Sizes for Multilevel Modeling. Methodol. Eur. J. Res. Methods Behav. Soc. Sci..

[21] D’Hondt E., Venetsanou F., Kambas A., Lenoir M. (2019). Motor Competence Levels in Young Children: A Cross-Cultural Comparison Between Belgium and Greece. J. Mot. Learn. Dev..

[22] Haga M., Tortella P., Asonitou K., Charitou S., Koutsouki D., Fumagalli G., Sigmundsson H. (2018). Cross-Cultural Aspects: Exploring Motor Competence Among 7- to 8-Year-Old Children from Greece, Italy, and Norway. Sage Open.

[23] Kaioglou V., Dania A., Venetsanou F. (2020). How Physically Literate Are Children Today? A Baseline Assessment of Greek Children 8–12 Years of Age. J. Sports Sci..

[24] Kechagia O., Katartzi E., Fotiadou E., Giagazoglou P. (2024). Prevalence of Motor Difficulties in Greek Preschoolers: The Effect of Gender, Age, BMI, Place of Residence, and Hand Preference. Alta. J. Educ. Res..

[25] Li M.H., Kaioglou V., Ma R.S., Choi S.M., Venetsanou F., Sum R.K.W. (2022). Exploring Physical Literacy in Children Aged 8 to 12 Years Old: A Cross-Cultural Comparison between China and Greece. BMC Public Health.

[26] Ellinoudis T., Evaggelinou C., Kourtessis T., Konstantinidou Z., Venetsanou F., Kambas A. (2011). Reliability and Validity of Age Band 1 of the Movement Assessment Battery for Children—Second Edition. Res. Dev. Disabil..

[27] Henderson S.E., Sugden D., Barnett A.L. (2007). Movement Assessment Battery for Children-2.

[28] Goodway J.D., Ozmun J.C., Gallahue D.L., Saracho O.N., Spodek B. (2013). Motor Development in Young Children. Handbook of Research on the Education of Young Children.

[29] Koo T.K., Li M.Y. (2016). A Guideline of Selecting and Reporting Intraclass Correlation Coefficients for Reliability Research. J. Chiropr. Med..

[30] Wuang Y.-P., Su J.-H., Su C.-Y. (2012). Reliability and Responsiveness of the Movement Assessment Battery for Children–Second Edition Test in Children with Developmental Coordination Disorder. Dev. Med. Child Neurol..

[31] Hua J., Gu G., Meng W., Wu Z. (2013). Age Band 1 of the Movement Assessment Battery for Children-Second Edition: Exploring Its Usefulness in Mainland China. Res. Dev. Disabil..

[32] Cools W., Martelaer K.D., Samaey C., Andries C. (2009). Movement Skill Assessment of Typically Developing Preschool Children: A Review of Seven Movement Skill Assessment Tools. J. Sports Sci. Med..

[33] Pinheiro J.C., Bates D.M. (2000). Mixed-Effects Models in S and S-PLUS.

[34] Enders C.K., Tofighi D. (2007). Centering Predictor Variables in Cross-Sectional Multilevel Models: A New Look at an Old Issue. Psychol. Methods.

[35] Cohen J. (1992). A Power Primer. Psychol. Bull..

[36] Hothorn T., Bretz F., Westfall P. (2008). Simultaneous Inference in General Parametric Models. Biom. J. Biom. Z..

[37] Kleiman E. EMAtools: Data Management Tools for Real-Time Monitoring/Ecological Momentary Assessment Data, Version 0.1.4; CRAN, 2021. https://cran.r-project.org/src/contrib/Archive/EMAtools.

[38] R Core Team (2026). R: A Language and Environment for Statistical Computing, Version 4.5.2.

[39] Gelman A., Carlin J. (2014). Beyond Power Calculations: Assessing Type S (Sign) and Type M (Magnitude) Errors. Perspect. Psychol. Sci..

[40] Nakagawa S., Schielzeth H. (2013). A General and Simple Method for Obtaining R^2^ from Generalized Linear Mixed-Effects Models. Methods Ecol. Evol..

[41] Blank R., Barnett A.L., Cairney J., Green D., Kirby A., Polatajko H., Rosenblum S., Smits-Engelsman B., Sugden D., Wilson P. (2019). International Clinical Practice Recommendations on the Definition, Diagnosis, Assessment, Intervention, and Psychosocial Aspects of Developmental Coordination Disorder. Dev. Med. Child Neurol..

[42] Piek J.P., Dawson L., Smith L.M., Gasson N. (2008). The Role of Early Fine and Gross Motor Development on Later Motor and Cognitive Ability. Hum. Mov. Sci..

[43] Ting L.H., Gick B., Kesar T.M., Xu J. (2024). Ethnokinesiology: Towards a Neuromechanical Understanding of Cultural Differences in Movement. Philos. Trans. R. Soc. B Biol. Sci..

[44] Bril B. (2018). Action, Movement, and Culture: Does Culture Shape Movement?. Kinesiol. Rev..

[45] Adolph K.E., Hoch J.E. (2019). Motor Development: Embodied, Embedded, Enculturated, and Enabling. Annu. Rev. Psychol..

[46] Huang C.-Y., Huang T.-Y., Koh C.-L., Yu Y.-T., Chen K.-L. (2024). The Movement Assessment Battery for Children Second Edition in Ages 3 to 6 Years: A Cross-Cultural Comparison for Children in Taiwan. Phys. Ther..

[47] Koh C.-L., Lee T.-M., Chen K.-L., Huang C.-Y. (2025). Cultural Differences in Motor Skills and Psychometric Evaluation of the MABC-2 in Taiwanese School-Aged Children. Sci. Rep..

[48] Kokštejn J., Musálek M., Tufano J.J. (2017). Are Sex Differences in Fundamental Motor Skills Uniform throughout the Entire Preschool Period?. PLoS ONE.

[49] Barnett L.M., van Beurden E., Morgan P.J., Brooks L.O., Beard J.R. (2010). Gender Differences in Motor Skill Proficiency from Childhood to Adolescence: A Longitudinal Study. Res. Q. Exerc. Sport.

[50] Barnett L.M., Telford R.M., Strugnell C., Rudd J., Olive L.S., Telford R.D. (2019). Impact of Cultural Background on Fundamental Movement Skill and Its Correlates. J. Sports Sci..

[51] Bronfenbrenner U., Morris P.A., Damon W., Lerner R.M. (2007). The Bioecological Model of Human Development. Handbook of Child Psychology.

[52] Raudenbush S.W., Willms J.D. (1995). The Estimation of School Effects. J. Educ. Behav. Stat..

[53] Newell K.M., Liu Y.-T., Mayer-Kress G. (2001). Time Scales in Motor Learning and Development. Psychol. Rev..

[54] Smith L.B., Thelen E. (2003). Development as a Dynamic System. Trends Cogn. Sci..

[55] Davids K., Glazier P., Araújo D., Bartlett R. (2003). Movement Systems as Dynamical Systems. Sports Med..

